# Rank Order Coding: a Retinal Information Decoding Strategy Revealed by Large-Scale Multielectrode Array Retinal Recordings^1^,^2^,^3^

**DOI:** 10.1523/ENEURO.0134-15.2016

**Published:** 2016-06-03

**Authors:** Geoffrey Portelli, John M. Barrett, Gerrit Hilgen, Timothée Masquelier, Alessandro Maccione, Stefano Di Marco, Luca Berdondini, Pierre Kornprobst, Evelyne Sernagor

**Affiliations:** 1 Biovision Team, Inria Sophia Antipolis Méditerranée, FR-06902, Sophia Antipolis, France; 2 Faculty of Medical Sciences, Institute of Neuroscience, Newcastle University, Newcastle-upon-Tyne NE2 4HH, United Kingdom; 3 INSERM, U968, Paris, F-75012, France; 4 Sorbonne Universités, UPMC Univ Paris 06, UMR S 968, Institut de la Vision, Paris, F-75012, France; 5 CNRS, UMR 7210, Paris, F-75012, France; 6 Present address: CERCO UMR 5549, CNRS – Université de Toulouse, F-31300, France; 7 NetS3 Laboratory, Neuroscience and Brain Technologies Dpt., Istituto Italiano di Tecnologia, Genova, Italy.

**Keywords:** ganglion cells, multielectrode array, population coding, rank order coding, retina

## Abstract

How a population of retinal ganglion cells (RGCs) encodes the visual scene remains an open question. Going beyond individual RGC coding strategies, results in salamander suggest that the relative latencies of a RGC pair encode spatial information. Thus, a population code based on this concerted spiking could be a powerful mechanism to transmit visual information rapidly and efficiently. Here, we tested this hypothesis in mouse by recording simultaneous light-evoked responses from hundreds of RGCs, at pan-retinal level, using a new generation of large-scale, high-density multielectrode array consisting of 4096 electrodes. Interestingly, we did not find any RGCs exhibiting a clear latency tuning to the stimuli, suggesting that in mouse, individual RGC pairs may not provide sufficient information. We show that a significant amount of information is encoded synergistically in the concerted spiking of large RGC populations. Thus, the RGC population response described with relative activities, or ranks, provides more relevant information than classical independent spike count- or latency- based codes. In particular, we report for the first time that when considering the relative activities across the whole population, the wave of first stimulus-evoked spikes is an accurate indicator of stimulus content. We show that this coding strategy coexists with classical neural codes, and that it is more efficient and faster. Overall, these novel observations suggest that already at the level of the retina, concerted spiking provides a reliable and fast strategy to rapidly transmit new visual scenes.

## Abstract (precis)

How a population of retinal ganglion cells (RGCs) encodes the visual scene remains an open question. Going beyond individual RGC coding strategies, results in salamander suggest that the relative latencies of a RGC pair encode spatial information. Thus, a population code based on this concerted spiking could be a powerful mechanism to transmit visual information rapidly and efficiently.

## Visual Abstract

**Figure:**
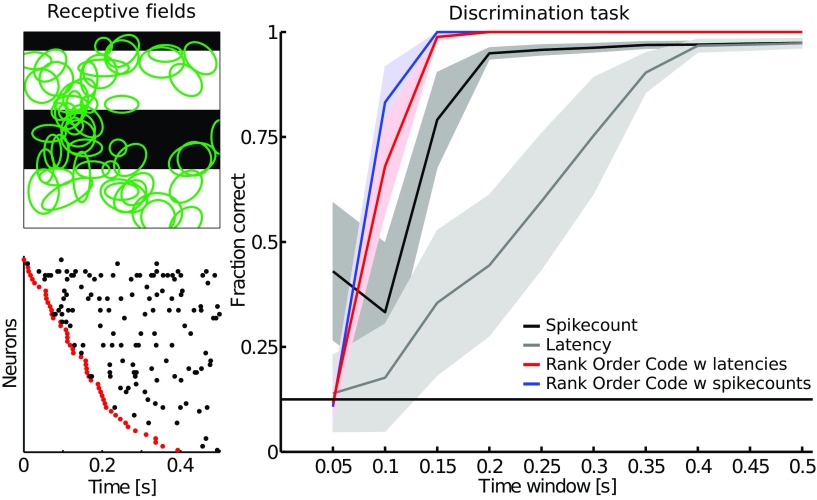

## Significance Statement

How the retina encodes the visual environment remains an open question. Using a new generation of large-scale high-density multielectrode array, we show that in large populations of mammalian retinal ganglion cells (RGCs), a significant amount of information is encoded synergistically in the concerted spiking of the RGC population. Thus, the RGC population response described with relative activities, or ranks, provides more relevant information than classical neural codes, such as independent spike count- or latency-based codes. In particular, and for the first time, we show that the wave of first stimulus-evoked spikes (WFS) across the whole population reliably encodes and rapidly transmits information about new visual scenes. This strategy of WFS could also apply to different sensory modalities.

## Introduction

Understanding information processing in the nervous system by exploring the neural code is a major challenge (Rieke et al., 1997). In the visual system, many questions remain open about how spike trains generated by retinal ganglion cells (RGCs) encode and convey information about the visual environment. Greschner et al. (2006) showed that information can be read-out from simple response features, such as the spike count, the latency of the first spike event, or the latency between different spike events. But simple coding strategies, such as spike count-based coding, are insufficient and more information-rich codes, such as spike-timing, that take into account the precise timing of occurrence of the spikes of individual RGCs are necessary to match behavioral performance (Jacobs et al., 2009).

Beyond the individual RGC coding strategies, the concerted spiking of a pair of RGCs, e.g., relative latencies of some RGC pairs, can encode spatial information in the salamander retina (Gollisch and Meister, 2008). In that paper, the authors suggested that “a population code based on differential spike latencies could be a powerful mechanism to rapidly transmit new visual scenes”. Otherwise stated, this amounts to considering the structure of the global concerted spiking pattern, i.e., the relative activities.

Among the algorithms available to read out concerted spiking patterns (Rieke et al., 1997), a classical one is the rank-order code (ROC) strategy, where the information is not coded in the precise timing of spikes for each input, but rather in the relative order in which the neurons fire (Gautrais and Thorpe, 1998; Thorpe et al., 2001). This coding strategy was established in the context of ultrafast visual categorization by considering that the human visual system can analyze and classify a new complex scene in <200 ms (Thorpe et al., 1996; Kirchner and Thorpe, 2006; Crouzet et al., 2010). The ROC strategy has computational advantages, such as robustness and fast processing, compared to classical spike count- and latency-based independent coding strategies (VanRullen et al., 2005). Therefore, by looking at the relative latency pattern, the ROC scheme may represent a strategy to access synergistically encoded information, i.e., information available in the population response that is not available when considering RGC responses individually. These advantages of ROC were highlighted using simplified retina models (VanRullen and Thorpe, 2001). However, to our knowledge, this has never been investigated experimentally.

In this study, we investigated whether the relative activities of a large RGC population might be a mechanism for encoding visual information in the mammalian retina. To this aim, we recorded the simultaneous activity from hundreds of mouse RGCs in response to flashing gratings with varying phases (as in Gollisch and Meister (2008)) and also with varying spatial frequencies. The RGCs were simultaneously recorded with the Active Pixel Sensor CMOS Multi-Electrode Array consisting of 4096 electrodes (4096 APS CMOS MEA) spanning an active area of 2.67 × 2.67mm (Berdondini et al., 2009, Maccione et al., 2014). These experiments led us to the three main following observations.

First, contrary to what has been found in salamander (Gollisch and Meister, 2008), in the mouse retina we did not observe any tuning of the relative latencies to the onset of the stimuli of individual RGC pairs, regardless of whether these cells were of the ON, OFF, or ON-OFF type. Inspection of the raster plots of all RGCs we recorded suggests that this lack of latency tuning may stem from strong spontaneous background activity, which is common in the mammalian retina. However, when considering the global relative activity pattern, we show that the wave of first stimulus-evoked spikes (WFS) is tuned to the grating phase.

Second, we found that a significant amount of information is encoded synergistically in the population response. Thus, the RGC population response described with relative activities might provide efficient coding capabilities. Using a Bayesian framework, we compared the coding performance of WFS (read-out with a ROC) with a correlated spike count code (ROC with spike counts) and classical spike count- and latency-based codes in a discrimination task that consisted of identifying the correct phase from a set of RGC responses.

Finally, we show that relative activities are more efficient than classical independent codes by comparing the discrimination performance with increasing size of the RGC population, and faster by varying the length of the observation window after the stimulus onset.

## Materials and Methods

In this paper, we present results from two retinal datasets, D1 and D2, with simultaneous recordings performed with the 4096 APS CMOS MEA, involving 764 and 649 RGCs respectively (D1: 39-day-old and D2: 52-day-old C57Bl/6 mouse). We had initially performed similar experiments using a conventional 60-channel MEA and pooled the data from several retinas (Dataset D3: 9 retinas, 258 RGCs). Mice of both sexes were used.

All experimental procedures were approved by the UK Home Office, Animals (Scientific Procedures) Act 1986.

### Stimulus design

The stimuli used in this study were modeled on those used by Gollisch and Meister (2008). In their study, the authors used square-wave gratings of varying phase and with a 300 μm bar width, which is 2.5 times the average RGC receptive field (RF) size in salamander. Here, the stimuli were 32 square wave gratings with four spatial frequencies and eight phases. Considering an average mouse RF of 200–300 μm, the bar widths used were 1600, 800, 400, and 200 μm in order to be close to the 2.5-fold ratio. As 1° = 30 μm on the mouse retina (Remtulla and Hallett, 1985), the four spatial frequencies correspond to 0.009, 0.018, 0.037, and 0.075 cycles per degree (cpd). We will use the notation mcpd to represent cpd values in the 10^−3^ range. For each spatial frequency, we define eight phases φ by applying to the gratings a shift of 1/4 × the bar width, ie, in phase angle φ ∈ {0, 45, 90, 135, 180, 225, 270, 315}°. The 32 stimuli are sorted by frequencies: stimuli 1–8 (9 mcpd), 9–16 (18 mcpd), 17–24 (37 mcpd), and 25–32 (75 mcpd). Each stimulus was presented 150 times in randomized blocks of 32 stimuli. Stimuli were flashed for 0.5 s followed by a uniform gray mask flashed for 1 s.

For the dataset D1, only the first 105 trials were considered in the analysis.

### High-resolution photostimulation and large-scale RGCs electrophysiological recordings

Datasets D1 and D2 presented here consist of the light-evoked responses of hundreds of adult mouse RGCs, which were simultaneously recorded using the 4096 APS CMOS-MEA platform (Biocam 4096, 3Brain GmbH; Maccione et al., 2014).

Animals were dark-adapted overnight prior to retinal isolation. On the day of the experiment, the mouse was killed by cervical dislocation, eyes were quickly enucleated and placed in artificial cerebrospinal fluid (aCSF) containing the following (in mm): 118 NaCl, 25 NaHCO_3_, 1 NaH_2_ PO_4_, 3 KCl, 1 MgCl_2_, 2 CaCl_2_, 10 glucose, and 0.5 l-glutamine, equilibrated with 95% O_2_ and 5% CO_2_. The retina was isolated from the eyecup and flattened, RGC layer facing down, onto the active area of the CMOS-MEA chip. Throughout recording, retinas were maintained at 32°C and perfused with aCSF at a rate of 1 ml/min. All surgical procedures were performed under dim red light and the room was maintained in darkness throughout the experiment.

Pan-retinal RGCs responses to visual stimuli were recorded using the 4096 APS CMOS-MEA platform integrated with a custom built high-resolution photostimulation system. The photostimulation system is based on a DLP video projector (“lightCrafter”, Texas Instruments), and was designed to project visual stimuli with micrometer spatial resolution over the entire retina and at sub-millisecond precision. Briefly, retinas were prepared and maintained on BioChips 4096S+ (3Brain GmbH). These CMOS-MEAs provide an array of 64 × 64 simultaneously recording electrodes over an active area of 2.67 × 2.67 mm and an overall plain area of 6 × 6 mm used to flatten the retina on the chip, ensuring good contact between the tissue and the electrodes. The platinum electrodes are 21 × 21 μm in size (42 μm pitch). Full-array recordings were performed at a sampling frequency of 7.06 kHz/electrode and a trigger signal generated by the photostimulation was simultaneously sampled at the same frequency in order to precisely synchronize the delivery of the light stimuli with the electrophysiological responses recorded from the RGCs. The total area covered by the light patterns is 664 × 664 pixels and each light-pixel covers 4 × 4 μm^2^ of the chip active area. Neutral density filters (ND 4, mean luminance 1.72 μW/cm^2^) were used to control the amount of light falling on the retina. Large-scale electrophysiological data from the 4096 electrodes were analyzed using a spike detector (Quantile-based event detection, Maccione et al., 2014; Muthmann et al., 2015) and single-unit spikes were sorted using the T-Distribution Expectation-Maximization algorithm in Offline Sorter (Plexon). Sorted units that had a reasonable amount of spike waveforms in relation to the recording length (∼>0.1 spikes/s) were then verified by visual inspection of the found clusters in the 2/3D principle component feature space (well separated clusters), calculated ISIs (>refractory period), and waveforms (different shapes) in the Offline Sorter GUI. Due to the high density of electrodes, the same units were sometimes detected on multiple electrodes. These redundant units were removed by comparing coincident spikes between neighboring units. Briefly, for each unit, spikes occurring within ±2 frames (1 frame = 1/7.06 ms) were detected in all units on the four closest electrodes and marked. This was done for all units, and then units with more than 5% coincident spikes were iteratively removed, such that for each coincident group only the one with the largest spike count was retained. We tested several thresholds but 5% seemed like a good compromise. Indeed, it is extremely unlikely that different units would repeatedly and consistently fire together within a window as brief as 700 ns (and because of the mosaic arrangement of RGC subtypes, it is unlikely to find responses originating from distinct RGCs, with different kinetics, within 40 µm from each other).

### RGCs electrophysiological recordings with conventional 60-MEAs

The dataset D3 consists of data pooled from nine mouse retinas (C57BL/6 mice aged 19–46 postnatal days) where the light-evoked responses of RGCs were recorded using a conventional 60-channel indium tin oxide MEA (60MEA200/30iRITO; Multichannel Systems). We presented the stimuli using a 6.5 inch LCD monitor (640 × 480 pixels, 60 Hz refresh rate), focused onto the RGC layer using a pair of lenses (Edmund Optics) and a 2× objective on an Olympus IX-71 inverted microscope. Stimuli were generated in MATLAB (MathWorks) and controlled using Psychotoolbox (Brainard, 1997; Pelli, 1997; Kleiner et al., 2007). Each monitor pixel covered an area of 23.333 × 23.333 μm^2^, so the four bar widths correspond to spatial frequencies of 10, 20, 40, and 80 mcpd. Retinas were prepared for recording using the same method as for APS CMOS-MEA experiments. Extracellular signals were acquired using an MEA1060-Inv amplifier, digitized, and sampled at 25 kHz by an MC_Card data acquisition card and recorded using MC_Rack (MultiChannel Systems). Action potentials were extracted offline in MC_Rack using a voltage threshold set at 6.5–8 standard deviations (SD) below the signal recorded on each channel during a baseline recording taken at the start of each experiment, before the retina was placed on the MEA. Spike sorting was done for all channels using the same procedure as for data recorded on the APS CMOS-MEA.

### RGCs selection and classification

Before the main flashing gratings stimulation, we applied two sequences for cell selection and classification purposes.

One of the sequences consisted of 15 min of randomly flickering (10 Hz) checkerboard (100 μm square) black or white stimuli. For each cell which had an average spike rate across the entire checkerboard stimulation >0.5 Hz, the spike trains were reverse correlated to the stimulus (spike triggered average; Chichilnisky, 2001), yielding an average 3D volume in space and time that triggers the cell to spike: the estimated RF. We considered the 2D-spatial component of the 3D RF at the time when the absolute value of the RF reaches its maximum. A custom blob-detection script in MATLAB (MathWorks) was used to select the 2D RFs that were well estimated. This approach yielded the selection of 764 RGCs for dataset D1, and 649 RGCs for dataset D2.

The other sequence consisted of full-field light stimulation with 60 repetitions of alternating homogeneous 2-s-white, 2-s-black stimuli. We estimated each unit’s instantaneous firing rate by convolving its spike train with a Gaussian (SD= 25 ms). We then computed a Bias Index (Carcieri et al., 2003) that measures the relative amplitude of the ON and the OFF responses. This index ranges from −1 for pure OFF responses to 1 for pure ON responses. We used this bias index to classify the cells into: OFF cells (−1<bias index<−0.33), ON-OFF cells (−0.33<bias index<0.33), and ON cells (0.33<bias index<1). For the dataset D3, any unit firing fewer than 30 spikes to the full-field stimulus was rejected (assuming a responding unit should have at least one spike per trial). Responses to white noise were not recorded in these retinas, so instead responsive units were detected using the Rayleigh test for non-uniformity of circular data on a subset (25/150) of the responses to the second-largest gratings (which were found by visual inspection of rasters to evoke the largest responses). Blocks containing trials used for detecting responsive units were excluded from further analysis.

### Spearman’s rank correlation coefficient

The Spearman rank correlation coefficient ρ is a nonparametric measure of statistical dependence between two variables. Applied here for a spike train of size *n* neurons, the *n* latencies of the first spikes *A*_i_ and *B*_i_ related to two different stimuli are converted to ranks *a*_i_, *b*_i_, and ρ is computed as the Pearson correlation coefficient *r* between the ranks:

(1) $$\text{ρ}=r_{a_{i},b_{i}}=\frac{\mathrm{cov}(r_{a_{i}},r_{b_{i}})}{\sigma_{a_{i}}\sigma_{b_{i}}}.$$

Identical latencies are assigned tied ranks and ρ is computed using the standard formula:

(2) $$\rho =1-\frac{6\left(\sum_{i=1}^{n}{\left(a_{i}-b_{i}\right)}^{2}+\overset{}{\underset{}{\sum }}\text{cf}\right)}{n(n^{2}-1)}\;with\;cf=\frac{m\left(m^{2}-1\right)}{12}\;,$$

where *cf* denotes a correction factor computed for each tied rank and *m* denotes the number of observations tied to a particular rank. As this correlation coefficient is measured on the ranks of spikes, this measure can be interpreted as a measure of how different are the ranks of the first occurring spikes driven by the two different stimuli: ρ = 1 for identical ranked lists and ρ = −1 for opposite ranked lists.

### Partial information decomposition

To quantify the amount of synergy contained in RGC population responses, we calculated the partial information decomposition (PID) for RGC pairs (Williams and Beer 2010). We chose PID for two reasons. First, it is asymmetric in that it quantifies mutual information between one random variable and an ensemble of random variables, making it a natural fit for experiments where we record responses of multiple neurons to a single stimulus. Second, unlike many other synergy measures used in the neuroscience literature, it is guaranteed to be non-negative and is able to measure synergy and redundancy simultaneously (Timme et al., 2014).

The idea behind PID is to decompose information provided by an ensemble of random variables *R* (e.g., responses of individual neurons) about another variable *S* (e.g., a stimulus) into the information provided by each variable individually, by each subset of variables, and by the whole ensemble. The full derivation of the PID is available from Williams and Beer (2010), but the calculation for the two-variable case is described below with the help of the partial information diagram in Figure 3*A*. The two inner circles represent the information carried by each individual variable about the stimulus:

(3) $$I(S;R_{i})=\underset{s}{\sum }\underset{r_{i}}{\sum }p(s,r_{i})\;\log_{2}\frac{p(s,r_{i})}{p(s)p(r_{i})}\text{ for }i=1,2.$$

Where the two circles overlap is the redundant information between the two variables. To calculate the redundancy, the specific information provided by each variable *R_i_* about a particular stimulus value *s* is first calculated as the Kullback–Leibler divergence between the distribution of *R_i_* conditioned on *s* and the marginal distribution of *R_i_*, i.e.:

(4) $$I(S=s;R_{i})=D_{KL}(R_{i}|S=s||R_{i})=\underset{r_{i}}{\sum }p(r_{i}|S=s)\log_{2}\frac{p(r_{i}|S=s)}{p(r_{i})}.$$

(The specific information is not explicitly expressed as a Kullback–Leibler divergence by Williams and Beer (2010), but the equivalence can be shown trivially by applying Bayes’s rule and basic logarithmic identities to their formula.) The redundancy is then the expectation over the stimulus distribution of the minimum specific information provided by either variable about each stimulus value, i.e.:

(5) $$\text{Red}(S;R_{1},R_{2})=\underset{s}{\sum }p(s)\underset{i=1,2}{\min}I(S=s;R_{i}).$$

The unique information carried by each variable is the mutual information between that variable and the stimulus less the redundant information:

(6) $$\text{Unq}(S;R_{i})=I(S;R_{i})-\text{Red}(S;R_{1},R_{2}).$$

The outer ellipse in Figure 2*A* represents the mutual information between the pair and the stimulus:

(7) $$I(S;R_{1},R_{2})=\sum_{s}\sum_{r_{1}}\sum_{r_{2}}p(s,r_{1},r_{2}){\text{log}}_{2}\frac{p(s,r_{1},r_{2})}{p(s)p(r_{1},r_{2})}.$$

Finally, the area of this ellipse not covered by the redundant or unique information is the synergistic information:

(8) $$\text{Syn}(S;R_{1},R_{2})=I(S;R_{1},R_{2})-\text{Unq}(S;R_{1})-\text{Unq}(S;R_{2})-\text{Red}(S;R_{1},R_{2}).$$

Substituting the equation for *Unq*(*S*;*R*_i_) into Equation 8 reveals the advantage of the PID over more intuitive measures of synergy, such as the redundancy–synergy index (RSI; used by Schneidman et al., 2011):

(9) $$\text{RSI}(S;R_{1},R_{2})=I(S;R_{1},R_{2})-I(S;R_{1})-I(S;R_{2}),$$

(10) $$\text{RSI}(S;R_{1},R_{2})=\text{Syn}(S;R_{1},R_{2})-\text{Red}(S;R_{1},R_{2}).$$

That is, the RSI is the PID synergy less the PID redundancy. A positive RSI is often taken to mean synergistic coding and a negative RSI redundant, but an RSI close to zero could mean anything from independent coding to a code that comprises equal parts synergistic information and redundant information with no independent information. Because we were interested in detecting synergy regardless of the nature of the remaining information, the PID was the more natural fit.

The PID can be defined similarly for larger ensembles, but the complexity of the corresponding partial information diagrams and the resulting expressions become excessively complex extremely quickly as the number of variables increases. Additionally, the more neurons are included in the ensemble, the higher the dimensionality of the underlying probability distributions and the more data is required to estimate them accurately and precisely (note that this limitation applies to all synergy measures based on mutual information, not just PID). For these reasons, we decided to restrict our analysis to the two-variable case, i.e., pairs of neurons.

We took each *R_i_* as the number of spikes fired by the *i*th neuron of a pair during the presentation of the stimulus. We calculated the PID for every pair of neurons that was unique up to ordering: that is, if the PID for a pair (*i,j*) was calculated, we did not calculate the PID for the pair (*j,i*). Due to the long presentation times (500 ms), we deemed it unnecessary to include any of the period immediately following the stimulus, as 500 ms is sufficient to capture the entire response of all but the most sustained of cells.

To correct for bias introduced by limited sampling of the data, each of *D_KL_*(*R_i_|S=s‖R_i_), I*(*S;R_i_*), and *I*(*S;R_1_,R_2_*) was separately bias corrected using the subsampling method of Gollisch and Meister (2008). Briefly, after obtaining an estimate using the whole data set of *N* trials, the data is randomly partitioned into halves, thirds, and so on, and new estimates calculated for each of these partitions. We fit a second-degree polynomial to the estimate as a function of the number of partitions: the intercept of this polynomial corresponds to the estimate one would obtain with infinite samples and is taken as an unbiased estimate of the true value. We also attempted to apply the PID to continuous response variables, such as first spike latency and whole spike trains, but were unable to find a sufficiently accurate and unbiased estimator of the underlying entropies and so those results are not reported here.

### Discrimination task

To quantify the performance of the relative activities in encoding stimulus information, we used a discrimination task. Based on RGC responses, the discrimination task consists of identifying the phase φ ∈ {0, 45, 90, 135, 180, 225, 270, 315}° among the eight gratings of a given spatial frequency. We used a classical supervised Bayesian classifier allowing different codes to be tested within the same formalism: the independent spike count code, the independent latency code, the WFS (ROC based on the latencies), and a correlated spike count code (ROC based on the spike counts).

From the available trials, one-half are randomly chosen as training set for each stimulus and the responses from the remaining trials are the testing set, corresponding to the unknown stimulus $\overset{¯}{\varphi }$. For each $\overset{¯}{\varphi }$, we find an estimate $\overset{˜}{\varphi }$ using the a maximum *a posteriori* criterion:

(11) $$\overset{˜}{\varphi }=\underset{\varphi }{\text{argmin}}\left\{-\log(P(\varphi |r_{\overset{¯}{\varphi }}))\right\},$$

where $r_{\overset{¯}{\varphi }}$ represents the set of responses from the tested phase. We used Bayes’s theorem to estimate *P* (φ|*r*) from the response distribution *P* (*r*|φ), which depends on the code chosen. For each stimulus $\overset{¯}{\varphi }$ tested, 150 different configurations of training set and test set were randomly chosen. Each time the Bayesian classifier was run to guess the phase $\overset{˜}{\varphi }$. Results were stored in a 8 × 8-confusion matrix *M* (*M*($\overset{˜}{\varphi },\;\overset{¯}{\varphi }$)) that was incremented after every classification. Each column of *M* represents the results over all configurations when a given phase $\;\overset{¯}{\varphi }$ was tested. If the maximum lies along the diagonal, then the image has been correctly decoded in a plurality of configurations. To quantify the performance, we estimated the fraction of correct predictions as the mean of the diagonal of the confusion matrix. The fraction of correct predictions lies on the interval [0, 1]. If $\;\overset{˜}{\varphi }$ is equal to $\;\overset{¯}{\varphi }$ for all $\overset{¯}{\varphi }$ tested in all trials, the fraction of correct predictions will be 1.

Four coding strategies are evaluated in this paper: (1) the spike count code, where *r* is the average number of spikes within the presentation time of the stimulus, when each neuron is considered as independent; (2) the latency code, where *r* is the latency of the first spike after the stimulus onset, in which case the response probability was estimated using a kernel density estimation (Gaussian function, σ=0,01 s); (3) the ROC based on the WFS, where *r* is the rank of the latency time stimulus onset for each neuron (named ROC with latencies), which can be directly obtained from estimating the relative ordering between all pairs of RGCs. In that case, for an RGC pair (*i*, *j*), the response distribution is defined by:

(12) $$P(r_{\left(i,j\right)}|\varphi )=C\underset{T}{\sum }H(L_{i}^{T}-L_{j}^{T}),$$

where the sum is over trials *T* of the training set, $L_{i}^{T}$ is the latency of neuron *i*, *C* is a normalization factor and:

(13) $$H(s)=\{\begin{array}{c} 0\text{ if }s\leq 0,\\ 1\;\text{otherwise}. \end{array}$$

(4) One could argue that the differences observed between the classical independent codes and the WFS may only stem from the correlations taken into account in the ROC scheme. Therefore we also included a coding strategy where the spike counts are used to rank the cells instead of the latencies (named ROC with spike counts), using the same methods as in (3). This can be related to a joint correlated spike count code.

Using this approach, the fraction of correct predictions is shown in Figure 5 for the different coding schemes and as a function of the frequency of the gratings. To investigate the effect of the size of the RGC population on the discrimination performance and the variation of the discrimination performance across time, only neural responses related to the 18mcpd gratings were considered. To compute the variation of the performance with the number of RGCs (Fig. 6), the fraction of correct predictions was estimated and averaged over 100 randomly chosen RGC subsets (cross-validation) ranging from 2 to 600 RGCs amongst the whole available RGC population. To compute the variation of the performance across time (Fig. 7), the fraction of correct predictions was estimated using an observation window that ranged from 0.05 to 0.5 s after the stimulus onset.

## Results

We present the results from two datasets obtained with the 4096 APS CMOS MEA (D1 with 764 RGCs and D2 with 649 RGCs). Initially, we performed similar experiments using conventional 60-channel MEAs and reached the same conclusions as for D1 and D2 by pooling the data from several retinas (D3: 9 retinas, 258 RGCs). However, datasets from individual retinas recorded with the 60-channel MEA did not produce significant results. The recording capabilities of the 4096 APS CMOS MEA allow us to simultaneously record from hundreds of RGCs in the same retina, yielding results with much more robust statistics. Assuming there are ∼4000 RGCs/mm^2^ (not including displaced amacrine cells; Rodriguez et al., 2014), or 0.004 RGCs/µm^2^, we estimate that each electrode-pixel area (measuring 42 ×42 µm, or 1764 µm^2^) can potentially record from a maximum of ∼7 RGCs. We record on average from 1 to 2 units per electrode-pixel area, which amounts to 14–29% of all theoretically available RGCs. This provides a huge step forward compared to what has been achieved with earlier recording platforms, enabling us to acquire a much clearer picture of how concerted spiking patterns across a large RGC population encode information about the stimulus. Despite small variability between preparations, the overall pattern of results obtained by the different techniques is the same, thus suggesting that the WFS is a powerful strategy for fast information transfer.

### Retinal responses are noisy but carry synergistic information

**Figure 1.**
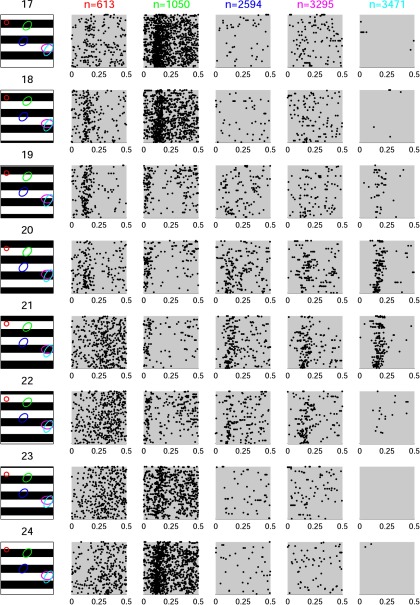
Typical RGC responses of the dataset D1 to flashed gratings of spatial frequency 37 mcpd and different phases. Colored ellipses superimposed on grating images show the estimated receptive fields of the chosen RGCs. For each RGC chosen, 105 repetitions recorded with the 4096 APS CMOS MEA are plotted from 0 s (stimulus onset) to 0.5 s. We found no RGC exhibiting a clear latency tuning to the grating phase. However, a clear modulation of the RGC spike count with the grating phase can be observed for some cells.

Typical RGC responses from the dataset D1 to flashing gratings with different spatial phases are illustrated in Figure 1. Contrary to previous reports in salamander (Gollisch and Meister, 2008), we found no RGC exhibiting a clear latency tuning to the grating phase. However, there is a clear modulation of the RGC spike count with the grating phase. However, despite that clear link between the spike count and grating phase, substantial levels of spontaneous activity appear to blur the temporal precision of the responses to the preferred stimuli in most cells (Fig. 1).

**Figure 2.**
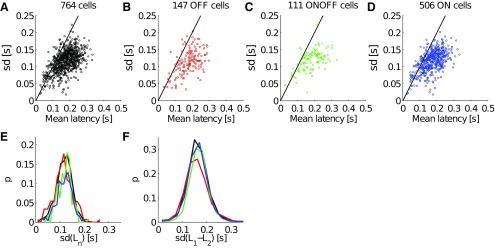
Latency variability. ***A***–***F***, Considering stimulus 17 (φ = 0°, 37mcpd). ***A***, The SD is plotted as a function of the mean latency over the 105 repetitions, for all 764 RGCs of the dataset D1, for (***B***) only the 147 OFF cells, for (***C***) only the 111 ON-OFF cells, or for (***D***) only the 506 ON cells (see Materials and Methods for the classification method). The black line corresponds to a SD that is equal to the mean latency. This shows the considerable variability of individual latencies. ***E***, The probability distribution of the individual latency SD for all cells (black), OFF cells (red), ON-OFF cells (green), and ON cells (blue). ***F***, The probability distribution of the SD of latency difference for all cell pairs (black), OFF cell pairs (red), ON-OFF cells pairs (green), and ON cell pairs (blue).

To estimate the overall reproducibility of the RGC responses, we plotted the SD versus the mean latency of the first spike for individual RGC responses over 105 trials of the first phase of the 37 mcpd gratings considering all 764 RGCs (Fig. 2*A*), or separating OFF cells (Fig. 2*B*), ON-OFF cells (Fig. 2*C*), and ON cells (Fig. 2*D*; see Materials and Methods). Surprisingly, all cells showed large variability in the latency of their first spike with a SD comparable to the mean. Within each cell type, the mean latency was variable but this variability was qualitatively similar in different cell types. These similarities in RGC responses are striking even when comparing the probability distributions of the SD (Fig. 2*E*). Thus, here the reproducibility of RGC responses to several presentations of the same stimulus seems to be quantitatively low, and therefore these latencies may not be an accurate indicator of the stimulus content. Similar results were obtained for D2 (data not shown).

Even if the latency of individual cells is noisy, i.e. the SD is large, perhaps the difference between the latencies of cell pairs (*L*_1_–*L*_2_) is more reliable, as shown by Gollisch and Meister (2008). In other words, the SD of the latency differences may be significantly smaller. We computed and plotted the probability distributions of the SD of latency differences for all cell pairs (black), OFF cell pairs (red), ON-OFF cell pairs (green), and ON cell pairs (red; Fig. 2*F*; dataset D1). Here again, the latency differences of RGC subpopulations seem to share the same variation across repeated presentations of the same stimulus. Thus, this rules out the possibility that there may be subsets of neurons in which the absolute relative latency is highly repeatable. Moreover, by comparing Figure 2, *E* and *F*, one could argue that the SD of the latency differences may be on average even larger than, or at least equal to, those of the individual latencies. This demonstrates that the latency differences of cell pairs are not an accurate indicator of the stimulus content either. Similar results were obtained for D2 (data not shown).

**Figure 3.**
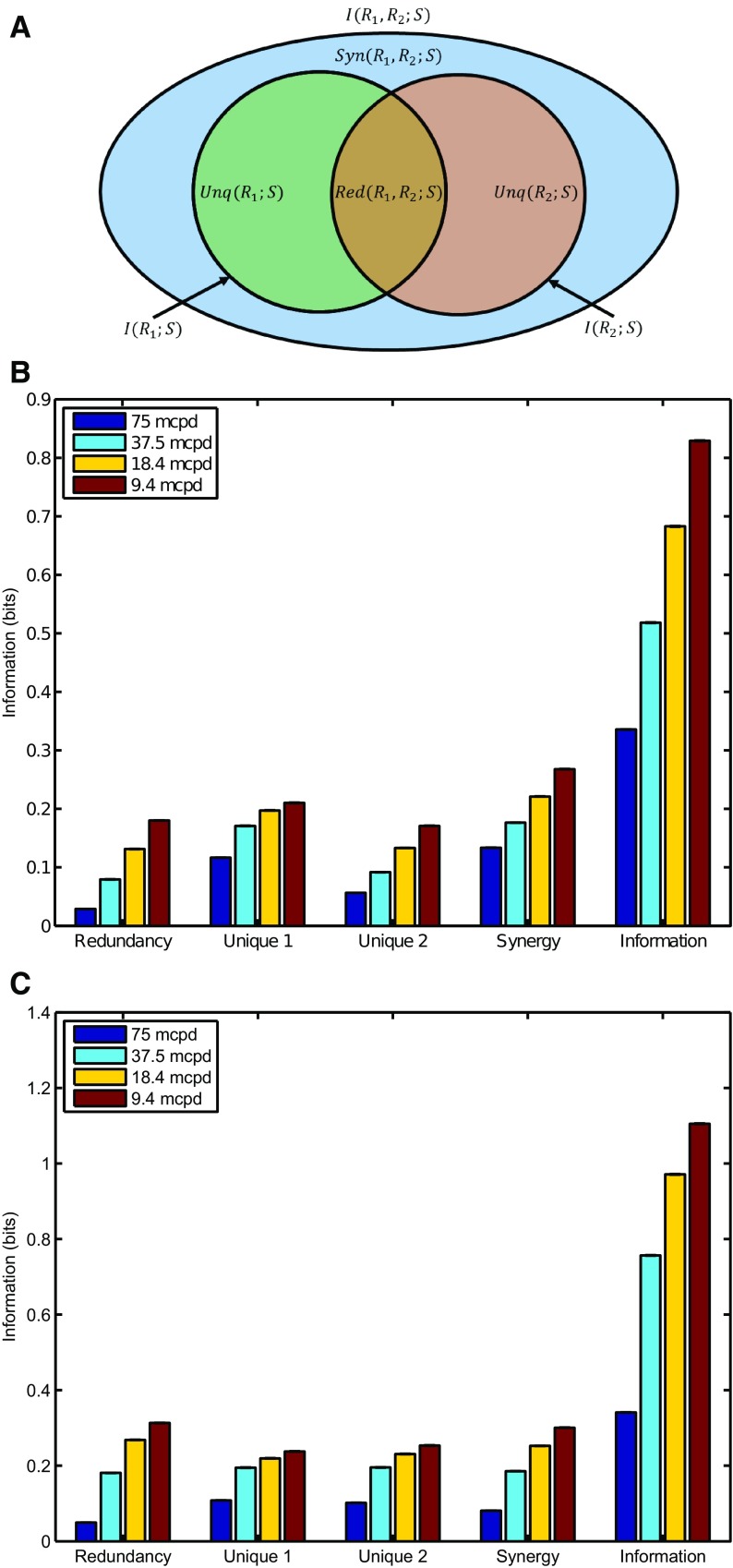
***A***, Partial information diagram for two variables, based on Williams and Beer (2010), their Figure 1. The two inner circles represent the mutual information between two variables, *R*_1_ and *R*_2_, considered separately, and a third variable *S*. Where they overlap is the redundant information; where they do not is the unique information provided by each. The outer ellipse represents the mutual information between the pair (*R*_1_,*R*_2_) and *S*. The area not covered by the inner circles is the synergistic information. Decomposition of the information using PID for (***B***) dataset D1 and (***C***) dataset D2. The histograms show the amount of redundant, unique, and synergistic information for the four different spatial frequencies (9, 18, 37, and 75 mcpd). Error bars show standard error on the mean (SEM), but due to the large number of pairs sampled they are too small to be visible.

As modulation of the RGC spike count with the grating phase is nevertheless conspicuous (Fig. 1), we performed a PID (see Materials and Methods) to quantify the amount of redundant, unique, and synergistic information available in the spike counts (Fig. 3*B*,*C*; dataset D1and dataset D2, respectively). This analysis shows that a considerable portion of the available information carried by the spike trains is synergistic; suggesting that the relative activities, i.e. the concerted spiking pattern of the entire RGC population, carries information that is not available in the spiking of individual neurons. Shuffling the responses to each stimulus of one neuron of each pair relative to the other had a negligible effect on the PID (data not shown), suggesting that the synergy does not arise due to noise correlations. This analysis also suggests that although the noise level (spontaneous activity) may impair the reliability of the responses in individual RGCs, more reliable results are achieved when considering multiple RGC responses simultaneously rather than when treating individual RGC responses separately.

### Accessing the synergistic information with the relative activities

Although the PID results suggested there was synergy in RGC pair spiking responses, the limitations of the PID (see Materials and Methods) prevent us from using it to answer how much synergy there is in larger populations or other response features, such as the timing of spikes. To address these questions indirectly, we investigated whether the WFS, which takes the relative activities of the entire RGC population into consideration, could be a plausible alternative indicator of the stimulus content. So here, the synergistic information conveyed by the WFS refers to the mean response properties of the neurons, i.e. to signal correlations in the response rather than to noise correlation.

To quantify the differences between the WFS obtained with gratings of different phases, we used the Spearman rank correlation coefficient ρ (see Materials and Methods). This measure can be interpreted as a distance between two ranked lists: ρ = 1 for identical ranked lists and ρ = −1 for ranked list that are opposite.

**Figure 4.**
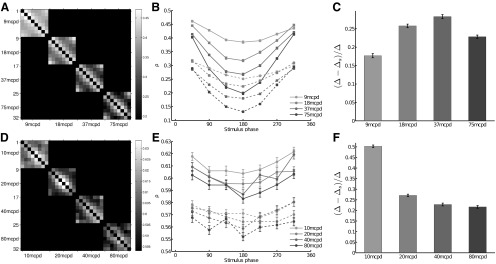
Distance between WFS evoked by different stimuli. ***A***, Confusion matrix showing the mean Spearman correlation coefficient ρ computed for all possible stimuli pairs, across all the trials of the dataset D1. It can be interpreted as a measure of how different are the ranks of the first stimulus-evoked spikes related to two different stimuli: ρ = 1 for identical ranked lists and ρ = −1 for opposite ranked lists. Periodic patterns appear, which can be related to phase differences. ***B***, For each spatial frequency, the variations of ρ(0, φ)|{φ=45...315}, where φ are the other gratings differing with their phases, are plotted. Continuous lines stand for ρ computed across the trials. Dashed lines stand for ρ computed using shuffled trials. The more the phase changes, the more the ranked emitted spikes are different. Shuffling the trials decreases this modulation. Error bars show SEM. ***C***, Quantification of the effect due to shuffling the trials observed in ***B*** as a relative difference between ρ(0,45) and ρ(0,180) in normal (Δ) and shuffled (Δs) condition (see Results for details). Shuffling the trials leads to a loss of ρ up to 30%. ***D***, ***E***, ***F***, Same analysis as in ***A***, ***B***, ***C***, using the dataset D3. Periodic variation of ρ as a function of the phase can be seen but not as clear as in dataset D1. Error bars show SEM.

Figure 4*A*–*C* shows the Spearman rank correlation analysis for the dataset D1 (similar results were obtained for D2, data not shown). Figure 4*A* shows the mean rank correlation ρ between responses recorded from two stimuli φ, computed across all trials, between all stimuli pairs φ*_i_* and φ*_j_* sharing the same spatial frequency, i.e. ρ(φ*_i_*, φ*_j_*). This representation shows periodic patterns matching the phase differences. Given a spatial frequency and the grating with phase 0° as a reference, one can plot the variations of ρ(0, φ)|{φ=45...315}, where φ are the phases of the other gratings. Results are shown in Figure 4*B* (continuous lines): ρ(0, φ) is high for phases near 0° and decreases for phases φ = 90° to 180°. The ρ varies cyclically with the phase of the gratings and this effect is even stronger for high spatial frequencies, suggesting that the WFS is tuned to the phase of the grating and that it is a good indicator of the stimulus content. One could assume that even if the individual cell latencies may have some trial-to-trial variability, these variations could be positively correlated in cells recorded simultaneously, leading to a preservation of the relative activities from trial to trial. This hypothesis can be assessed by artificially destroying the noise correlation by pairing RGC responses belonging to different trials. By pairing RGC responses shifted by one trial, Gollisch and Meister (2008) observed a loss of up to 20% of the mutual information. Here, we paired RGC responses by randomly shuffling the trials, resulting in an overall loss of correlation. Results are shown in dashed lines in Figure 4*B*. WFSs are less distinct from each other, but the shuffling of trials does not completely impair the information contained in the WFS, as the tuning to the phase is still visible. To quantify the loss related to the shuffling of trials for each frequency, we compared the average difference across trials between ρ(0,45) and ρ(0,180) denoted by Δ and the same quantity when trials are shuffled, denoted by Δ*s*. Figure 4*C* shows (Δ−Δ*s*)/Δ as a function of the grating frequency. Shuffling the trials leads to a loss of ρ up to 30%.

Figure 4*D*–*F* shows the Spearman rank correlation analysis for the dataset D3. Both datasets D1 and D3 show similar periodic variation of the distance as a function of the phase. However, this effect is less clear for dataset D3. In this particular data set, the spikes are ranked within each recorded retina (responses of RGCs belonging to different retinas are not paired). Thus, even if in total there are 258 RGCs, in practice only a few of them encode the stimulus content simultaneously. For dataset D1, the use of 4096 APS CMOS MEA provides a huge improvement in deciphering the concerted spiking pattern of a large RGC population because here 764 cells are simultaneously taken into consideration.

### Relative activities provide efficient coding capability

**Figure 5.**
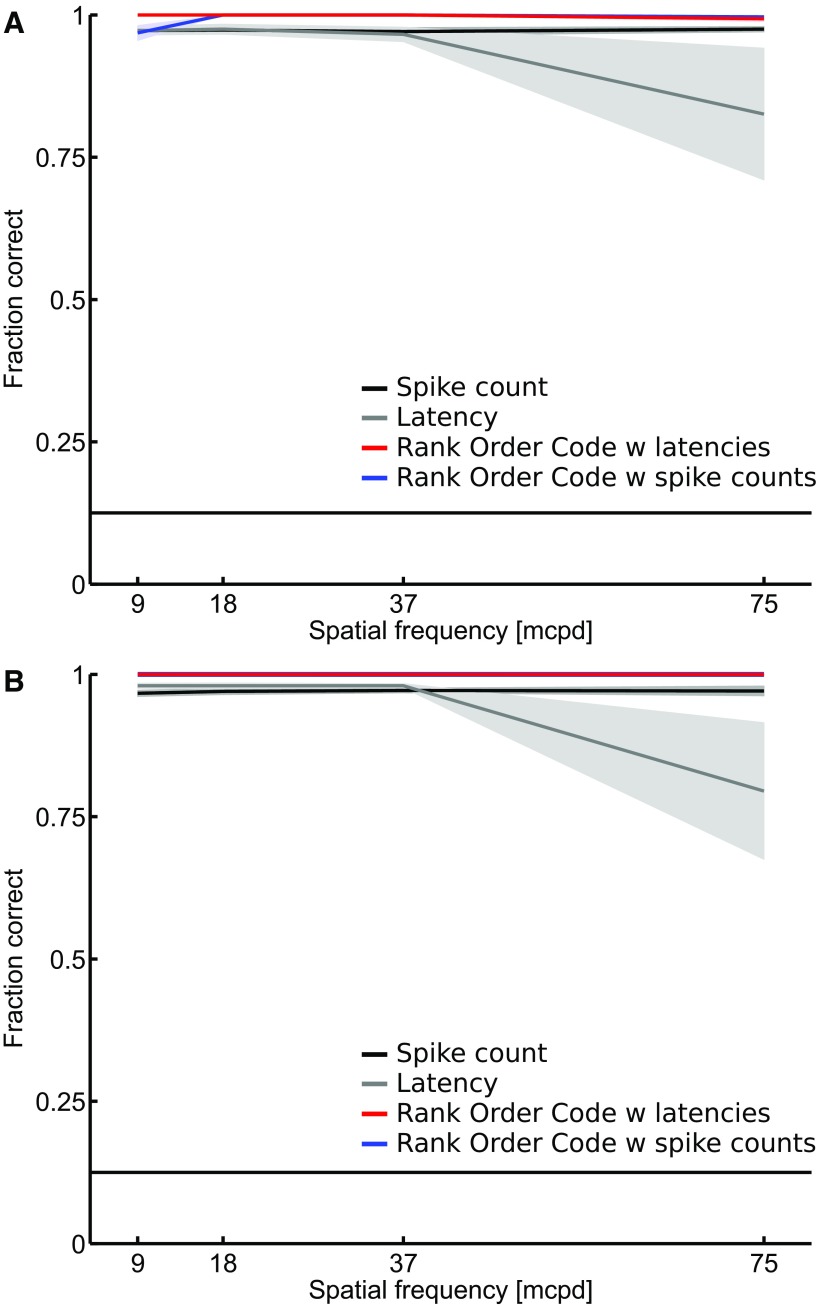
Discrimination performance of the spike count, the latency, and the ROC decoders. The fraction of correct identifications is plotted as a function of the spatial frequency for the spike count code (black), the latency code (gray), the ROC with latencies (red), and the ROC with spike counts (blue). ***A***, All 764 RGCs of the dataset D1 and (***B***) all the 649 RGCs of the dataset D2 were used in this analysis. Results show that all the decoders perform well in this task (close to 1, maximal value). The horizontal line indicates chance level. Shaded areas show SEM.

To quantify the coding capability of the relative activities, we considered a discrimination task consisting of identifying which of the eight gratings is represented in the RGC population response for a given spatial frequency (see Materials and Methods). Figure 5 shows a comparison of the fraction of correct identifications for the independent spike count code (black), the independent latency code (gray), the ROC with latencies (red), and the ROC with spike counts (blue). All 764 RGCs of dataset D1 (Fig. 5*A*) and 649 RGCs of dataset D2 (Fig. 5*B*) were used in this analysis. Results show that all the decoders perform well in this task (close to 1, maximal value), even if the latency decoder seems to slightly lose performance at the highest spatial frequency. Note that although the individual RGC responses were not precise in time (large SD values; Fig. 2*A*), the sum of the information contained in the spiking of individual RGCs was sufficient to perform well in this task. This may be due to the large number of RGCs considered with different response patterns and the low spatial complexity of the stimuli used in this task. The ROC with spike counts (correlated spike count) and the ROC with latencies (WFS) still appear to outperform the classical decoders, demonstrating that the relative activities efficiently encode for spatial information about the stimulus.

### Relative activities enable efficient transmission of visual information with only few neurons

**Figure 6.**
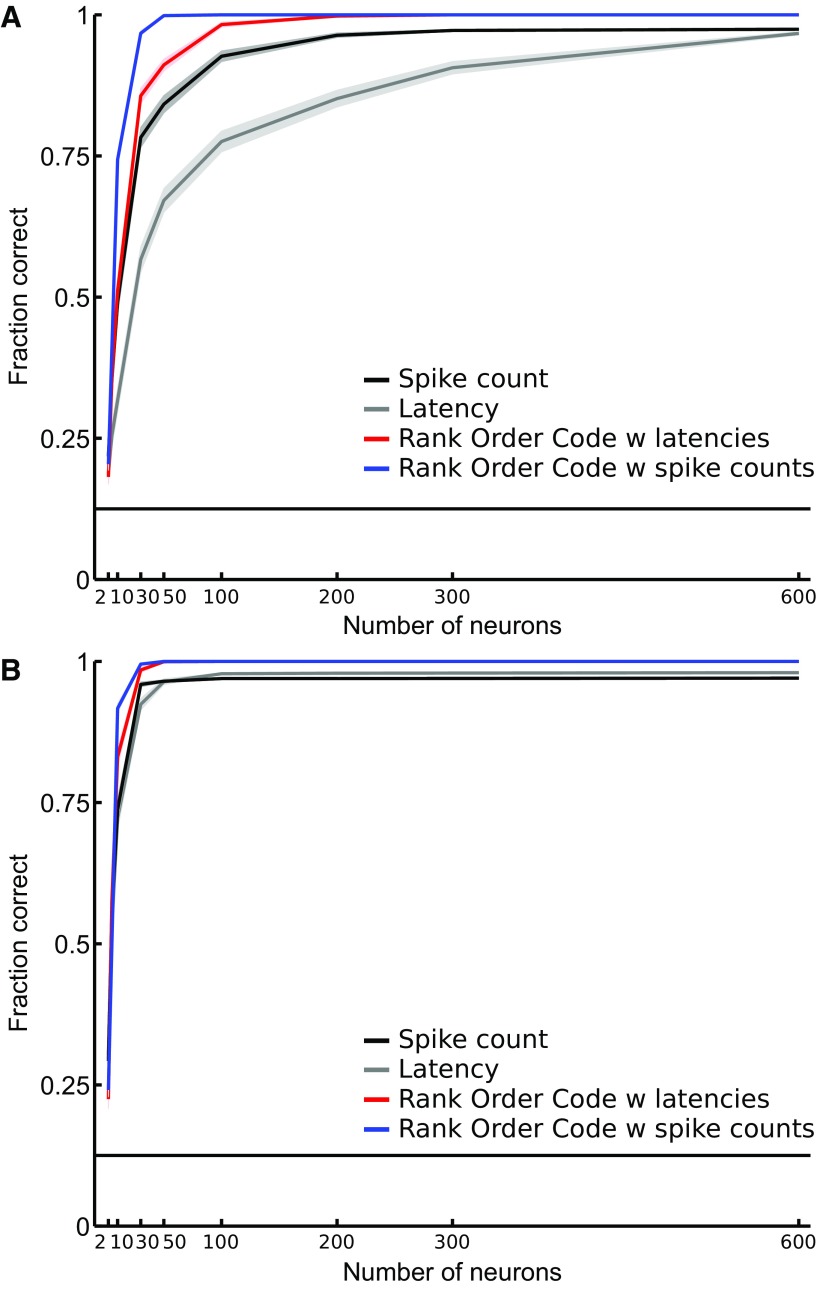
Discrimination performance as a function of the number of RGCs. The fraction of correct identifications is plotted for the spike count code (black), the latency code (gray), and the ROC with latencies (red), and the ROC with spike counts (blue), as a function of the number of neurons. ***A***, Responses of the dataset D1 related to stimuli 9–16 (18 mcpd) are used in this analysis. From a population size of 30 RGCs and higher, the ROC with latencies tends to perform better than the latency decoder. ***B***, Analysis on the responses of the dataset D2 related to the same stimuli as in ***A***. The horizontal line indicates chance level. Shaded areas show SEM.

One may wonder whether the large number of RGCs may obscure more subtle differences in the coding efficiency of the spike count code, the latency code, the ROC with latencies, and the ROC with spike counts. To address this question, we investigated how the decoders’ performances vary with the size of the RGC population. We performed the discrimination task with increasing numbers of RGCs and considering only gratings of 18 mcpd spatial frequency. At this spatial frequency and when all the RGCs are taken into consideration, all four decoders performed equally well, with a score ≥0.9 (Fig. 6). Figure 6 shows the evolution of the fraction of correct identifications as a function of the number of RGCs, from 2 to 600 RGCs, for the dataset D1 (Fig. 6*A*) and the dataset D2 (Fig. 6*B*). As expected, all four decoders perform better when the number of RGCs increases. However, in Figure 6*A*, the ROC with spike counts and the ROC with latencies both rapidly outperform the classical spike count and latency decoders. To illustrate the benefit of taking correlations in the response into account, let us focus on the ROC with latencies in Figure 6*A*. It reaches a score of 0.8 with only 30 neurons. The independent latency decoder needs 300 neurons to reach the same 0.8 score. Thus, to reach 80% accuracy level like a correlated latency code (WFS) does with 30 cells, one would need 300 independent cells, i.e. 10 times more independent cells. Even if the overall performances are better than for dataset D1, similar results were obtained for dataset D2 (Fig. 6*B*).

### Relative activities enable fast transmission of visual information

**Figure 7.**
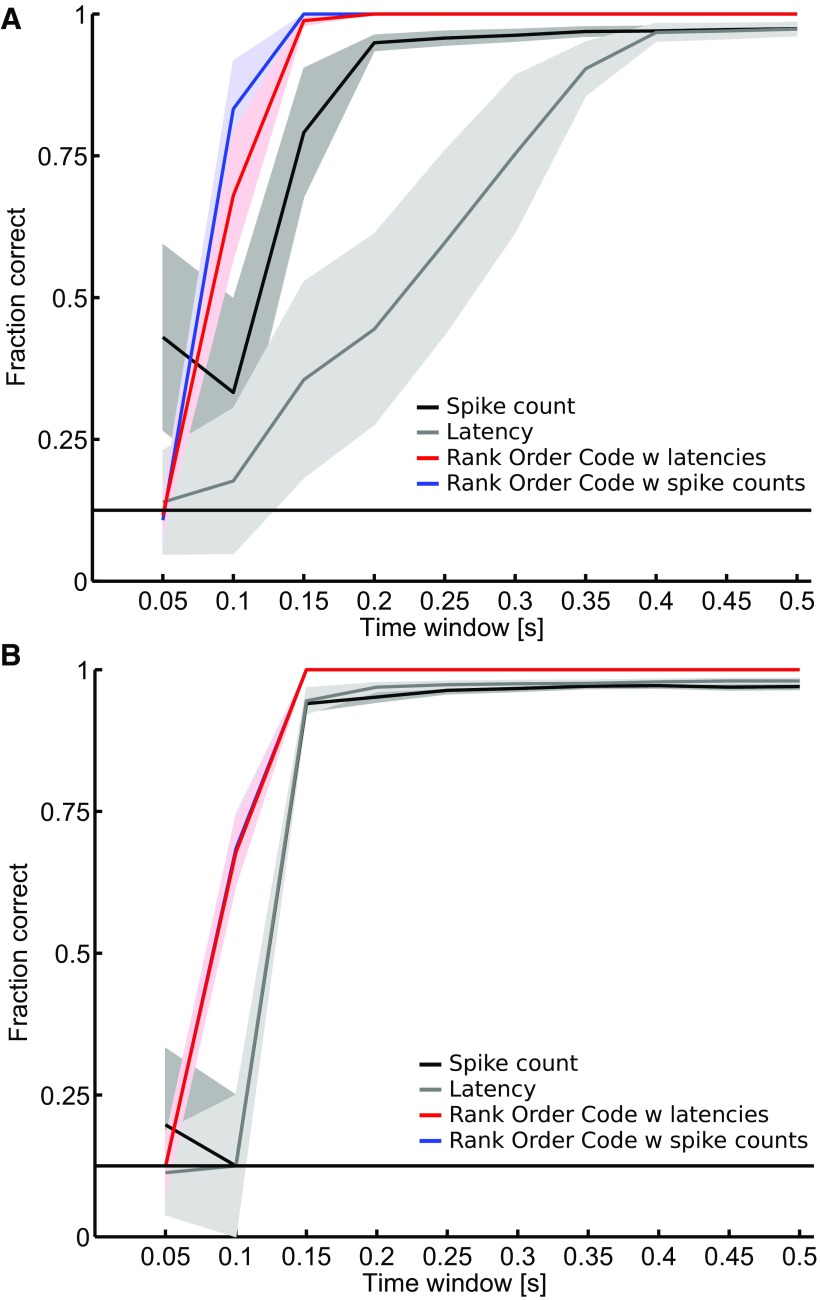
Discrimination performance as a function of the time window after the stimulus onset. The fraction of correct identifications is plotted for the spike count code (black), the latency code (gray), the ROC with latencies (red), and the ROC with spike counts (blue) as a function of the length of the observation window. This time window varied from 0.05 to 0.5 s after the stimulus onset. Responses of (***A***) the dataset D1 and (***B***) the dataset D2, related to stimuli 9–16 (18 mcpd) are used in this analysis. ROC with latencies decoder rapidly outperforms the latency decoder and reaches its maximal performance within 0.15 s after the stimulus onset. ***B***, The curve of the ROC with spike counts is hidden by the ROC with latencies. The horizontal line indicates chance level. Shaded areas show SEM.

Finally, we investigated how fast each of the four coding strategies can transmit information by computing the fraction of correct identifications as the length of the observation window varied from 0.05 to 0.5 s after the stimulus onset. Responses to the 18 mcpd spatial frequency gratings were used in this analysis and the results are shown in Figure 7*A* for dataset D1, and in *B* for dataset D2. Overall, the performance of all four decoders increases with the length of the observation window. In Figure 7*A*, the independent spike count and the independent latency decoders respectively need 0.2 and 0.4 s after the stimulus onset to reach their maximal performances. Once again, the ROC with spike counts and the ROC with latencies decoders rapidly outperform the two independent decoders and they reach their maximal performance within 0.15 s after the stimulus onset. So here, even though both ROCs and independent decoders are based on the same basic measure (latencies or spike counts), taking into account the correlation within the population significantly improves performance, enabling rapid transmission of the relevant information. Although the overall performances are better than for dataset D1, similar results were observed for dataset D2 (Fig. 7*B*).

## Discussion

Several coding strategies have been investigated by different groups using mostly artificial stimuli. Two main streams of thought have emerged: one considering RGCs as independent encoders, and another one considering them as synergistic encoders, i.e. when the relative activities in a RGC population contains information that is not available in the spiking of individual RGCs. Nirenberg et al. (2001) argued that RGCs encode information independently as they measured very little increase in mutual information between stimulus and response when taking into account correlations between RGCs versus considering them independently. However, as the same group notes in a later paper (Latham and Nirenberg 2005), synergistic information can exist in a system without pairwise correlations being important for decoding. Moreover, there is a growing body of evidence that when RGCs are considered as synergistic encoders, they carry complementary and more precise information about the stimulus.

Overall, our findings suggest that synergistic encoding of information in the relative activities of a neuronal population is a feature of RGC responses at the population level. Here, we used the PID (Williams and Beer, 2010) to directly quantify the amount of synergy in the RGC population response and found it to be a considerable fraction of the total information carried by pairs of neurons. Shuffling the data did not reduce the synergy, so noise correlations are unlikely to be the source. Therefore, how this synergy arises is unclear and remains an interesting topic for future work. It should be noted that, in the absence of noise correlations, the synergy defined in Equation 8 reduces to *Red*(*S*;*R*_1_,*R*_2_) – *I*(*R*_1_;*R*_2_), and thus is maximized as signal correlations go to zero (assuming fixed redundancy). This suggests a combinatorial code in which different cells encode orthogonal stimulus features. Possible examples include distinct cell types providing complementary information about the stimulus or cells with spatially separate receptive fields providing information about the spatial structure of the stimulus that is unavailable when considering individual neurons. As a simple example of the former, consider an ON cell that fires a single spike if and only if it sees a light increment in some part of its receptive field and an OFF cell that responds similarly to light decrements. Both cells have overlapping receptive fields. Imagine that both cells are illuminated by a uniform grey field that is replaced, with equal probability, by either a black field, a white field, or a black and white field split down the center of the two receptive fields (this example is similar to that used by Williams and Beer (2010), to illustrate the asymmetry of the PID). Either cell alone can distinguish one stimulus from the other two, but not the remaining two from each other (e.g. the ON cell fires to both the white and split fields but not the black field). Distinguishing all three stimuli requires both cell types and, according to PID, 21% of the information about the stimulus available in pair responses is synergistic, but the information lost by ignoring correlations in this system is exactly zero. Obviously, this example is not representative of real retinal coding, but rather serves to illustrate how synergy can arise through different cell types without informative pairwise correlations. The amount of synergy may also depend on the stimulus itself, with different stimulus classes lending themselves more or less well to synergistic encoding. Direction selectivity is an example of this. Imagine two direction-selective cells with perpendicular preferred directions that fire strongly to motion along their preferred direction, weakly or not at all to motion against this direction and moderately to motion perpendicular to it. Suppose we wish to distinguish bars moving in four perpendicular directions aligned with the two cell’s preferred directions. Both cells provide redundant information about which axis the bar is moving along. Additionally, each cell provides unique information about whether the bar is moving towards or against its preferred direction. This is all the information there is to be about the bar’s motion direction: unlike in the split fields example, there is no synergistic information, illustrating how different stimuli can affect the amount of synergy present. However, we cannot address the question of how the stimulus affects the amount of synergy with the type of stimulus (square-wave gratings) used here.

Having demonstrated the existence of synergistic information in the population response, several strategies can be used to decode the relative activities. Assuming that the firing order is stimulus-specific, the simplest algorithm is the winner-take-all decoder (Barnden and Srinivas, 1993). In this decoder, for an incoming firing pattern across the entire RGC population, the decision of the classifier is determined by the RGC with the shortest latency. However, this decoder can be unreliable, especially if the timing of incoming spikes is variable, for instance when there is strong spontaneous activity (as observed in our recordings), or if spikes generated by different RGCs occur in very short succession, or even become completely synchronous. Another possibility is to consider the spatiotemporal patterns of all spikes within a given time window and to use the tempotron algorithm (Gütig and Sompolinsky, 2006). The tempotron consists of a single integrate-and-fire model neuron (IF) that receives inputs from the population of RGCs. Depending on the relative timing of the incoming spikes and on their synaptic weights (that are *a priori* determined; supervised algorithm), the summation of all the inputs will determine whether the IF neuron will fire or not. Thus, this model can classify the input spikes patterns into those that elicit a spike in the IF neuron, as well as those that do not trigger the IF neuron. The tempotron was used to analyze salamander retinal responses and was able to decode complex visual features (Gütig et al., 2013). The authors applied this decoding strategy to fast-OFF RGCs, using a total of only 41 pooled RGCs recorded from nine different retinas. However, how this coding scheme would behave with other RGC subtypes or with a mixture of RGC subtypes, and how performance will be affected by using a larger RGC population were left as open questions.

In the present study, we investigated in the mouse retina whether the relative latencies between neuron pairs could be a good indicator of the stimulus content, as shown by Gollisch and Meister (2008) for the salamander retina, but the outcome was negative. RGCs in the salamander retina exhibit lower levels of spontaneous activity (Gollisch and Meister, 2008) than in mouse (Fig. 1). Therefore salamander RGCs demonstrate high reproducibility in their latencies (especially for so-called fast OFF RGCs) to the onset of the same stimulus (with only a few milliseconds of latency SD), which may explain why the authors were able to detect fine-tuning of the absolute relative latencies between pairs of neurons. Unfortunately, the low reproducibility observed here in mouse RGC responses (Fig. 4) might have hidden fine-tuning of absolute relative latencies. One could also argue that those animals (salamander vs mouse) are different from an ecological and behavioral point of view and that their visual systems may have been tuned to fit their own ecological constrains.

Going further, we investigated whether the population response as a whole could be a better indicator of the stimulus. We have applied a simpler decoding strategy based on the ROC decoder (Thorpe et al., 2001), which can take latencies (ROC with latencies, WFS read-out) or spike counts (ROC with spike counts) as inputs, to a large, mixed RGC population (D1: 764 RGCs; D2: 649 RGCs), regardless of their specific functional subtypes. Here, the WFS is represented by the rank of the first stimulus-evoked spikes for each RGC. To assess the performance of the ROC decoder for the stimuli used in this work, we designed a discrimination task where the goal was to identify the phase of the gratings. We found that the ROC with latencies and spike counts decoders are able to perform the task better than the spike count- or the latency-based decoder (Fig. 5). Going a step further, we wondered how the size of the RGC population could impact the performance of each decoder in the discrimination task. This question is important since in a more naturalistic scenario, one could argue that local analyses of spatial structure based on fewer specialized cells will be required. To answer this question we performed the discrimination task using increasing numbers of RGCs (Fig. 6). Even if all decoders increase their performance with the number of RGCs, the ROC with latencies and spike counts tend to perform better than the classical independent decoders for populations of 50 RGCs or more. The difference in the effect of number of neurons on the WFS and individual latency codes in particular is consistent with the findings of Schwartz et al. (2012), who reported that, for large numbers of neurons, a latency code assuming independent neurons suffers greatly in performance compared to one that exploits the full correlation structure of the latencies.

Regardless of the RGC subtype and the level of spontaneous activity, one of the main conclusions is that the WFS robustly encodes sufficient information about spatial cues to succeed in this discrimination task. Because there is evidence that different RGC subtypes encode different features of the stimuli (van Wyk et al., 2006; Zhang et al., 2012), an interesting perspective would be to further investigate the specific role of each subpopulation of RGCs within the WFS. More generally, assuming that the functional and morphological characterization of RGCs is available, one could consider an ensemble of discrimination tasks and determine which subpopulations are relevant for each task.

ROCs convey visual information faster than classical coding strategies. This is what we observed by comparing the discrimination performances of the different decoders as a function of the duration of the time window after the stimulus onset (Fig. 7). Already at the retinal output level, we show that a simple decoder that exploits the relative activities allows the visual information to be extracted much faster than the classical decoders. These results are in line with previous studies which have suggested that the ROC scheme, initially based on the latencies, could be an efficient and fast strategy for processing visual information (Thorpe et al., 2001; Guyonneau et al., 2005; VanRullen et al., 2005; Masquelier and Thorpe, 2007). The relevance of the WFS for a whole RGC population read-out by a ROC has been investigated at the retinal level using a simulated RGC population (VanRullen and Thorpe, 2001). However, since we used multiple trials for the decoding as in Jacobs et al. (2009), one could argue that the direct link to the original concept in rapid single-trial classification tasks (Thorpe et al., 1996) is lost. Nevertheless, we reran the analysis using all-but-one cross-validation (hence each trial is decoded individually) and found the WFS (ROC with latencies) to be at least as good as (in one retina) or better than (in the other) the independent spike count code and in all cases better than the independent latency decoder. Figure 7 demonstrates that the rank format makes things easier for the classifier (discarding noise, but not signal). This is consistent with the idea that some of the trial-to-trial variability in the latencies is shared across cells. This kind of variability is detrimental to the independent code, but not to the ROC scheme.

Although our results demonstrate the power and efficiency of the ROC scheme, they give no hint as to how it might be implemented biologically. One has to ponder that a code based on the absolute relative latencies in the entire population should subsume the WFS code and, hence, could perform better. But to our knowledge, only mechanisms which are sensitive to a tight spike timing correlation, such as spike-timing-dependent plasticity, have been reported in the literature and could plausibly be able to read out the earliest firing inputs, i.e. here the WFS (Guyonneau et al., 2005; Masquelier et al., 2008). Decoding latency ranks could be done by biologically plausible mechanisms, such as shunting inhibition (Thorpe et al., 2001). To our knowledge, no one has ever proposed a mechanism to decode spike count ranks.

Nevertheless, one has to note that the ROC with spike counts tends to perform slightly better than the ROC with latencies (Figs. 5–7). For this particular task, it is highly possible that the information provided by the ROC with spike counts is superior to what the other codes investigated here are able to provide (but it may not be the case for more complex stimuli). Already in Figure 1, the modulation of the spike count across the stimuli is visible by eye in the raster plots, which is not the case for the latencies. Therefore, the information carried by the spike count would be less noisy than the information carried by latencies. Thus, even if taking into account correlations between neuron latencies (ROC with latencies) extracts more of the total information available in the latencies, the ROC with spike count wins over, because it there is more information in the firing rates to begin with. The most important point here is that those results are in line with previous studies where the functional significance of the concerted firing pattern has been investigated, for instance using a model of multineuron spike responses (Pillow et al., 2008). The authors showed that a read-out model that exploits the response correlation structure extracts 20% more information about the stimulus than a read-out model based on the independence assumption, and also preserves 40% more visual information than optimal linear decoding. Otherwise stated, if there are correlations in the firing patterns of a RGC population, it is beneficial to incorporate this structure in the read-out model.

We must remember that the stimuli used in our study are simple. All the four codes performed the discrimination task equally well. It may be that the discrimination task, as executed, is not sufficiently demanding to compare the potential performance of these codes thoroughly. The fine encoding provided by combinatorial codes might not be necessary or might not provide a lot more useful information about the stimuli than classical independent codes already do. Nevertheless, those combinatorial codes seem to do a better job at extracting information about the stimuli with small neural populations and short time windows (Figs. 6, 7). In future studies, it would be interesting to test those codes in a much more demanding discrimination task involving more complex stimuli.

How those codes would perform with a discrimination task involving stimuli with richer spatial content is an important open question and the answer may not be trivial. From Schwartz et al. (2012), when flashing black and white shapes onto salamander retinas, the authors reported that simple linear decoders, i.e. decoders based on independent spike train coding strategies, can only decode coarse stimulus properties such as the overall size or contrast. Thus, to perform high-fidelity discrimination, one needs nonlinear decoders that take correlations between RGC responses into account. So one could assume that in a discrimination task involving richer stimuli, independent coding schemes would perform less well than coding schemes that take into account correlations in the population responses. In other words, the ROC-based scheme, which considers the relative activities, would perform better than classical independent schemes in complex discrimination tasks.

Nevertheless, one could wonder whether the performance of the WFS represents a true timing code or is merely an artefact of rate coding. For example, one would intuitively expect a cell with a high stimulus-driven firing rate to fire its first spike following the stimulus sooner, on average, than a cell with a much lower stimulus-driven firing rate. We reran the discrimination analysis with jittered spike times (σ = 20 ms, data not shown), which should destroy timing information while preserving rate information, and saw no clear differences in WFS performance. Combined with the large amount of information available in correlated spike counts (i.e. the ROC with spike counts) here, this is consistent with (but does not prove) a latency code that arises as an epiphenomenon of rate coding. However, the debate between whether or not latency coding is an artefact of rate coding is an open question and a complete discussion of this is beyond the scope of this paper.

We are not arguing that there is only one reliable neural code. Indeed, there might be several concurrent, parallel streams of information sent from the retina to the brain, each encoding different stimulus features (Masland, 2012). Here we show that, in parallel with the classical individual spike count and individual latency codes, the codes based on relative activities, e.g. the WFS, also coexist and may encode reliable information about the visual scene. To our knowledge, our study represents the first experimental evidence that the relative activities and in particular the WFS, i.e. the first stimulus-evoked spikes across the whole RGC population, obtained by large-scale RGC population recordings are relevant, and our results suggest that the ROC scheme can be a powerful mechanism to encode and transmit visual information through visual pathways.

Because understanding how neurons fire with respect to one another is of fundamental importance for deciphering neural codes in sensory systems, our results on the WFS may have implications beyond retinal coding. In the olfactory system, the WFS and spike-timing in neuronal ensembles play an important role in information encoding (Shusterman et al., 2011; Smear et al., 2011). In the somatosensory system, it has been shown that the relative timing of the first spikes after stimulus onset contains rich information about the stimulus, such as the direction, the force, and the shape of the surface contacting the fingertip (Johansson and Birznieks, 2004). Similar observations have also been reported in the auditory system (de Charms and Merzenich, 1996; Chase and Young, 2007; Brasselet et al., 2012). All these observations reinforce the universality and power of the WFS, which represents a common denominator in various sensory modalities, conveying sufficient information for the encoding and fast transmission of relevant sensory information to the brain, allowing it to process and produce fast sensory-input driven appropriate responses.

## Synthesis

The decision was a result of the Reviewing Editor Tatyana Sharpee and the peer reviewers coming together and discussing their recommendations until a consensus was reached. A fact-based synthesis statement explaining their decision and outlining what is needed to prepare a revision is listed below. The following reviewers agreed to reveal their identity: Andrea Barreiro, Rava Azeredo da Silveira

The study "The wave of first spikes: a retinal information coding strategy revealed by large-scale multielectrode array recordings" discusses a possible population code in the context of extensive retinal recordings. Specifically, it compares a rank order code (ROC) in the population to the two more commonly considered codes based on spike counts and spike latencies. Two aspects of the work represent a refreshing and notable advance compared to earlier work. First, the data analysis is carried out on recordings of a population with several hundreds of neurons, a much larger figure than that in most previous studies. Second, the authors propose a neural coding strategy, the ROC, which is a true population code and which the authors show to be more robust than the code based on latency.

Two aspects of the work need to be addressed prior to publication. First, please the ROC code needs to compared with the spike and latency code in the presence of correlations. Second, Reviewer 2 points out that the code has been tested on 2 out of 3 datasets. It would be helpful to either add this analysis on the third dataset or explain why this is not possible/feasible to do.

Specific comments from Reviewers are attached below.

Reviewer 1:

1)The spike-count based code and latency code assume the neurons are independent, while the ROC presumably incorporates significant correlation information. If the authors are interested in investigating rate-based vs. timing based codes, wouldn't the fair comparison be with a correlated spike-count based code?

To put this another way: when decoding, both the spike-count and latency codes use a response vector of length N (N = number of cells) whereas the ROC code uses, at a minimum (see my comment below) a vector of length N(N-1)/2. Not surprising, that if you are allowed to sample 100-times more variables, you get better decoding.

To follow up on this: later the authors plot correct ID fraction vs. # cells required (pg. 18, first paragraph, and Figure 6); it would be interesting to see this comparison for a CORRELATED spike count code.

Alternatively, to ask (and this would just require re-plotting the results already shown here, no additional analyses): how many independent cells would be required to get the same accuracy of the WFS code: i.e. how many "extra cells" does a correlated code buy you?

2) Methodological questions:

pg. 7: Re: removing redundant units in spike sorting. Can you comment on the 5% threshold for removing redundant units. Isn't it possible for neighboring cells to share 5% coincident spikes?

pg. 8: Re: Selecting RGCs. How complete do you think your population is? i.e. what fraction of RGCs in the stimulated area are being included in the analysis? For a downstream target (say a simple cell in V1), what fraction of inputs are being included?

pg. 12: Some more details in describing the Bayesian classifier would be helpful. Specifically: I do not understand how the response distribution P(r | \phi) is defined for the ROC task. Is the definition of the response vector "the relative ordering between all pairs of RGCs"? I.e. a 0 or a 1 for each distinct pair. And if so, are those pairs then assumed to be independent?

pg. 15, second paragraph: It looks like the distribution of latency differences is what would be expected from the sum (or difference) of two independent random variables (i.e. if individual latencies were uncorrelated)

Var(r1 - r2) = Var(r1) + Var(r2): thus, if Var(r1) = Var(r2), then

Sqrt(Var(r1-r2)) ∼ 1.4 Sqrt(Var(r1))

That seems consistent w/ Fig. 2E,F.

3) Minor points, mostly having to do with language:

pg. 3, paragraph 1, sentence starting "Greshner et al. (2006) showed": use commas instead of "or" to list coding strategies.

Define "WFS" (i.e. "wave of first stimulus-induced spikes") in the main text. It is defined in the significance statement, but main text uses only the acronym WFS.

pg. 5, "Stimulus Design", first sentence: I suggest "modeled on those used by" instead of "inspired by"

pg. 5, "Stimulus Design", second line: Is "2,5" supposed to be "2.5"?

pg. 6, last line: there is a "2" that I think is supposed to be a superscript.

pg. 12, second line: I suggest "identifying the phase" instead of "finding what is the phase"

pg. 14-15, paragraph beginning "To estimate the overall temporal precision": I find the language here confusing. I think the authors are saying:

- each cell showed significant variability in the latency of its first spike (with a standard deviation comparable to the mean)

- within each cell type, the mean latency was variable

- this variability was qualitatively similar in different cell types.

If so, think this could be stated more simply.

If the authors then want to interpret these observations as saying something about "reproducibility of [cell] responses to several presentations of the same stimulus", they should do that as a summary sentence to the paragraph.

pg. 16, first line: "are" -> "may be" (authors have not yet demonstrated their claim)

pg. 17, first paragraph: "Figure 3C" should be "Figure 4C".

pg. 20: sentence beginning "Imagine that both cells are illuminated by": I think this is the same example as in Williams and Beers, should cite it.

pg. 23, sentence beginning "Here we show that": "in parallel of" should be "in parallel with" or "in parallel to"

pg. 23, sentence beginning "Our results on the WFS": I suggest rephrasing "Since understanding how neurons fire with respect to one another is of fundamental importance for understanding neural codes in other sensory systems, our results on the WFS have implications beyond retinal coding."

or something like that

Figures 5 and 6 would benefit from using a logarithmic axis for the "x" axis.

Figures 5 and 6: is "standard error of the mean" referring to the mean over "configurations of training set and test set" referred to on pg. 12?

Figure 6 legend, sentence beginning "From population size of 50": I think "better than the latency decoder" is meant to be "better than the spike count decoder".

Reviewer 2:

1. I have been wondering about the choice of measures employed to characterize the data, as well as about the relative consistency of these measures. This is true, in particular, for the choice and consistency of the PID. First, the PID relies on spiking rates (calculated over a long, 500 ms window), whereas the rest of the paper wants to emphasize synergy in the context of rank order. Second, all the synergy coming from noise correlation is, by definition, included in the synergistic term in the PID. More specifically, the coupling between the two neurons in the pair, in the redundancy term, is achieved through the minimum operation that appears in the third equation on p. 10; but the joint probability distribution of the response of the two neurons is not considered. Third, overall the choice of the PID is not very strongly motivated, especially since (as the authors point out themselves) it is laborious to use this quantity beyond pairs of neurons (and indeed the authors do not go beyond pairs). It would be good either to switch to a more intuitive or natural measure, or to motivate the PID more thoroughly and discuss the above points.

2. Figure 4B indicates that much of the information conveyed by the ROC comes from the mean response properties of the neurons, i.e., from signal correlations in the response, rather than from noise correlation. The authors touch upon this issue in a nice passage that they include in the Discussion, but it would be good to make it very clear that 'synergy', here, refers also, and possibly mostly, to signal correlations in the population.

3. Regarding Figs. 4B and 4E, the authors should also show the standard deviation across trials, so that one can estimate whether the modulation of the curves which are displayed are meaningful (e.g., in terms of decoding).

4. In the context of their decoding analysis, the authors examine the dependence on the population size. Already in the context of the analysis presented in Fig. 4, however, it would be good to quantify the effect of the population size.

5. Discrimination task: It seems to me that the discrimination task, as the authors pose it, is 'easy' in the sense that any code will perform it with low error rate provided the population is large enough. And the results of the analyses shown here also suggest that this is the case. Thus, I would be cautious not to overestimate the scope of Figs. 5 and 6. Could the authors discuss this point? In particular, how do the authors expect that the results in Figs. 5 and 6 may change if the discrimination task is made more difficult?

6. Comparing different codes, and Fig. 7: If the latency code includes signal and noise correlations, then it subsumes the ROC. If so, the latency code cannot perform worse than the ROC. If the latency code neglects some of the correlations, then of course it is possible that its performance be worse than the ROC. Still, I wonder why the ROC is so much faster than the latency code, as displayed in Fig. 7, since both codes rely on the first spike after stimulus onset. Is it because latency is less robust to noise than rank? Could the authors comment on the relative status of the two codes and on the discrepancy in their behavior as shown in Fig. 7?

7. Relevance of a rank code: Do the authors think of a rank code as a code which may actually be used in the brain? If so, can they speculate on how this fits more generally with visual processing and downstream read-out? Or do the authors think of the rank code as a caricature of a finer code used in the brain? Furthermore, what do the authors think of the discrepancy between Gollisch and Meister's results in salamander and their own results in mouse -- are different codes used in these different animals or are the latency code and the ROC both caricatures of a more involved, unknown code? It would be nice to see a discussion of these issues in the paper.

8. Methods: In the context of the discussion on p. 12-13, the authors should provide a more thorough description of how the population response probability densities are modeled, for their use in the decoding algorithm. In particular, how do the authors deal with finite sampling issues?

9. Early on in the paper, the authors mention three data sets, two of them using large-scale recordings (D1 and D2) and the third one using recordings of smaller populations (D3). In the remainder of the paper, only analyses of D1 and D3 are shown. Why is not D2 also used? It should be easy to add curves on all the graphs, corresponding to D2.

Secondary points:

a. It looks like Fig. 2F rules out the possibility that there may be subsets of neurons in which the relative latency is highly repeatable. If so, the authors should state this explicitly.

b. There are a few issues with references to figures:

- p. 9, penultimate line, and p. 10, last line: "Fig. 2A" should be replaced by "Fig. 3A";

- p. 16, line 15: "Fig. 3B" should be replaced by "Fig. 4B";

- Fig. 3A is not mentioned in the main text; it should be, or else moved to Methods;

c. Fig. 3B: the error bars are not visible.

d. All equations should be numbered.

e. Equation on p. 9: "N" should be replaced by "n" and the form of the normalization should be explained.

f. The notation in the second equation on p. 10 is not consistent and should be fixed.

g. Page 12, line 9: it looks like it is a maximum a posteriori criterion, not a maximum likelihood criterion.

h. Page 12, line 4: the subscript "i" is missing on "LT."

## References

[B1] Barnden J, Srinivas K (1993) Temporal winner-take-all networks: a time-based mechanism for fast selection in neural networks. IEEE Trans Neural Netw 4:844–853. 10.1109/72.248461 [TQ1][TQ2] 18276513

[B2] Berdondini L, Imfeld K, Maccione A, Tedesco M, Neukom S, Koudelka-Hep M, Martinoia S (2009) Active pixel sensor array for high spatio-temporal resolution electrophysiological recordings from single cell to large scale neuronal networks. Lab Chip 9:2644–2651. 10.1039/b907394a 19704979

[B3] Brainard D (1997) The Psychophysics Toolbox. Spat Vis 10:433–436. 9176952

[B4] Brasselet R, Panzeri S, Logothetis NK, Kayser C (2012) Neurons with stereotyped and rapid responses provide a reference frame for relative temporal coding in primate auditory cortex. J Neurosci 32:2998–3008. 10.1523/JNEUROSCI.5435-5411.2012 22378873 PMC6622010

[B5] Carcieri SM, Jacobs AL, Nirenberg S (2003) Classification of retinal ganglion cells: a statistical approach. J Neurophysiol 90:1704–1713. 10.1152/jn.00127.2003 12966177

[B6] de Charms RC, Merzenich MM (1996) Primary cortical representation of sounds by the coordination of action-potential timing. Nature 381:13. 8637597 10.1038/381610a0

[B7] Chase SM, Young ED (2007) First-spike latency information in single neurons increases when referenced to population onset. Proc Nat Acad Sci U S A 104:5175–5180. 10.1073/pnas.0610368104 PMC1829282 17360369

[B8] Chichilnisky EJ (2001) A simple white noise analysis of neuronal light responses. Network 12:199–213. 11405422

[B9] Crouzet SM, Kirchner H, Thorpe SJ (2010) Fast saccades toward faces: face detection in just 100 ms. J Vis 10(4):16 1-17. 10.1167/10.4.16 20465335

[B10] Gautrais J, Thorpe SJ (1998) Rate coding versus temporal order coding: a theoretical approach. Biosystems 48:57–65. 9886632 10.1016/s0303-2647(98)00050-1

[B11] Gollisch T, Meister M (2008) Rapid neural coding in the retina with relative spike latencies. Science 319:1108–1111. 10.1126/science.1149639 18292344

[B12] Greschner M, Thiel A, Kretzberg J, Ammermüller J (2006) Complex spike-event pattern of transient on-off retinal ganglion cells. J Neurophysiol 96:2845–2856. 10.1152/jn.01131.2005 16914608

[B13] Gütig R, Sompolinsky H (2006) The tempotron: a neuron that learns spike timing-based decisions. Nat Neurosci 9:420–428. 10.1038/nn1643 16474393

[B14] Gütig R, Gollisch T, Sompolinsky H, Meister M (2013) Computing complex visual features with retinal spike times. PLoS One 8:e53063 10.1371/journal.pone.0053063 23301021 PMC3534662

[B15] Guyonneau R, VanRullen R, Thorpe SJ (2005) Neurons tune to the earliest spikes through stdp. Neural Comput 17:859–879. 10.1162/0899766053429390 15829092

[B16] Jacobs AL, Fridman G, Douglas RM, Alam NM, Latham PE, Prusky GT, Nirenberg S (2009) Ruling out and ruling in neural codes. Proc Natl Acad Sci U S A 106:5936–5941. 10.1073/pnas.0900573106 19297621 PMC2657589

[B17] Johansson RS, Birznieks I (2004) First spikes in ensembles of human tactile afferents code complex spatial fingertip events. Nat Neurosci 7:170–177. 10.1038/nn1177 14730306

[B18] Kirchner H, Thorpe SJ (2006) Ultra-rapid object detection with saccadic eye movements: visual processing speed revisited. Vis Res 46:1762–1776. 10.1016/j.visres.2005.10.002 16289663

[B19] Kleiner M, Brainard D, Pelli D (2007) What’s new in Psychtoolbox-3? Perception 36 ECVP Abstract Supplement.

[B20] Latham PE, Nirenberg S (2005) Synergy, redundancy, and independence in population codes, revisited. J Neurosci 25:5195–5206. 10.1523/JNEUROSCI.5319-5304.2005 15917459 PMC6724819

[B21] Maccione A, Hennig MH, Gandolfo M, Muthmann O, Coppenhagen J, Eglen SJ, Berdondini L, Sernagor E (2014) Following the ontogeny of retinal waves: pan-retinal recordings of population dynamics in the neonatal mouse. J Physiol 592:1545–1563. 10.1113/jphysiol.2013.262840 24366261 PMC3979611

[B22] Masland RH (2012) The neuronal organization of the retina. Neuron 76:266–280. 10.1016/j.neuron.2012.10.002 23083731 PMC3714606

[B23] Masquelier T, Thorpe SJ (2007) Unsupervised learning of visual features through spike timing dependent plasticity. PLoS Comput Biol 3:e31 10.1371/journal.pcbi.0030031 17305422 PMC1797822

[B24] Masquelier T, Guyonneau R, Thorpe SJ (2008) Spike timing dependent plasticity finds the start of repeating patterns in continuous spike trains. PLoS One 3:e1377 10.1371/journal.pone.0001377 18167538 PMC2147052

[B25] Muthmann JO, Amin H, Sernagor E, Maccione A, Panas D, Berdondini L, Bhalla US, Hennig MH (2015) Spike detection for large neural populations using high density multielectrode arrays. Front Neuroinform 9:28 10.3389/fninf.2015.00028 26733859 PMC4683190

[B26] Nirenberg S, Carcieri S, Jacobs A, Latham PE (2001) Retinal ganglion cells act largely as independent encoders. Nature 411:698–701. 10.1038/35079612 11395773

[B27] Pelli D (1997) The VideoToolbox software for visual psychophysics: transforming numbers into movies. Spat Vis 10:437–442. 9176953

[B28] Pillow JW, Shlens J, Paninski L, Sher A, Litke AM, Chichilnisky E, Simoncelli EP (2008) Spatio-temporal correlations and visual signalling in a complete neuronal population. Nature 454:995–999. 10.1038/nature07140 18650810 PMC2684455

[B29] Remtulla S, Hallett P (1985) A schematic eye for the mouse, and comparisons with the rat. Vis Res 25:21–31. 3984214 10.1016/0042-6989(85)90076-8

[B30] Rieke F, Warland D, de Ruyter van Steveninck R, Bialek W (1997) Spikes: exploring the neural code. Boston: MIT. 10.1126/science.2063199 2063199

[B31] Rodriguez AR, de Sevilla Müller LP, Brecha NC (2014) The RNA binding protein RBPMS is a selective marker of ganglion cells in the mammalian retina. J Comp Neur 522:1411–1443. 10.1002/cne.23521 24318667 PMC3959221

[B32] Schneidman E, Puchalla JL, Segev R, Harris RA, Bialek W, Berry MJ (2011) Synergy from silence in a combinatorial neural code. J Neurosci 31:15732–15741. 10.1523/JNEUROSCI.0301-3009.2011 22049416 PMC3446770

[B33] Schwartz G, Macke J, Amodei D, Tang H, Berry IIM (2012) Low error discrimination using a correlated population code. J Neurophysiol 108:1069–1088. 10.1152/jn.00564.2011 22539825 PMC3424080

[B34] Shusterman R, Smear MC, Koulakov AA, Rinberg D (2011) Precise olfactory responses tile the sniff cycle. Nat Neurosci 14:1039–1044. 10.1038/nn.2877 21765422 PMC13348895

[B35] Smear M, Shusterman R, O’Connor R, Bozza T, Rinberg D (2011) Perception of sniff phase in mouse olfaction. Nature 479:397–400. 10.1038/nature10521 21993623

[B36] Timme N, Alford W, Flecker B, Beggs JM (2014) Synergy, redundancy, and multivariate information measures: an experimentalist’s perspective. J Comput Neurosci 36:119–140. 10.1007/s10827-013-0458-4 23820856

[B37] Thorpe SJ, Fize D, Marlot C (1996) Speed of processing in the human visual system. Nature 381:520–522. 10.1038/381520a0 8632824

[B38] Thorpe SJ, Delorme A, VanRullen R (2001) Spike based strategies for rapid processing. Neural Netw 14:715–726. 10.1016/S0893-6080(01)00083-1 11665765

[B39] VanRullen R, Thorpe SJ (2001) Rate coding versus temporal order coding: what the retina ganglion cells tell the visual cortex. Neural Comput 13:1255–1283. 11387046 10.1162/08997660152002852

[B40] VanRullen R, Guyonneau R, Thorpe SJ (2005) Spike times make sense. Trends Neurosci 28:1–4. 10.1016/j.tins.2004.10.010 15626490

[B41] Williams PL, Beer RD (2010) Nonnegative decomposition of multivariate information. arXiv 1004.2515v1

[B42] van Wyk M, Taylor W, Vaney D (2006) Local edge detectors: a substrate for fine spatial vision at low temporal frequencies in rabbit retina. J Neurosci 26:13250 10.1523/JNEUROSCI.1991-06.2006 17182775 PMC6675005

[B43] Zhang Y, Kim IJ, Sanes JR, Meister M (2012) The most numerous ganglion cell type of the mouse retina is a selective feature detector. Proc Natl Acad Sci U S A 109:E2391–E2398. 10.1073/pnas.1211547109 22891316 PMC3437843

